# Safety of Permissive Cardiotoxicity of Trastuzumab in Patients with Breast Cancer: A Systematic Review and Meta-Analysis

**DOI:** 10.1007/s12012-025-10037-z

**Published:** 2025-07-08

**Authors:** Amira Mohamed Taha, Ramez M. Odat, Wesam Abd El-Tawab Moawad, Sara Adel Abdelkader Saed, Basma Ehab Amer, Dang Nguyen, Dalal Salama Salem, Linh Tran, Loay Kassem

**Affiliations:** 1 Faculty of Medicine, Fayoum University, Fayoum, Egypt; 2 Faculty of Medicine, Jordan University of Science and Technology, Irbid, Jordan; 3 Faculty of Pharmacy (Girls), Al-Azhar University, Cairo, Egypt; 4 MARS Global, London, UK; 5 Department of Clinical Pharmacy, MOH, Cairo, Egypt; 6 Faculty of Medicine, Benha University, Benha, Egypt; 7 Massachusetts General Hospital, Corrigan Minehan Heart Center, Harvard Medical School, Boston, MA USA; 8 Clinical oncology department, Faculty of Medicine, Cairo University, Cairo, Egypt; 9 University of Health Sciences, Vietnam National University Ho Chi Minh City, Ho Chi Minh City, Vietnam; 10 Vietnam National University Ho Chi Minh City, Ho Chi Minh City, Vietnam

**Keywords:** Trastuzumab, Cardiotoxicity, Breast cancer, EF

## Abstract

Cardiotoxicity is a recognized adverse effect associated with anti-HER2 therapies. The Trastuzumab-mediated cardiac dysfunction is not dose-dependent and lacks ultrastructural changes, allowing potential recovery after a few months. There is no consensus on the management of patients who develop cardiotoxicity. This meta-analysis aims to assess the safety of the permissive cardiotoxicity of Trastuzumab in patients with breast cancer. We searched PubMed, Cochrane Library, and Embase up to October 2023 for retrospective or prospective studies that investigated the safety of Trastuzumab in patients with breast cancer who continued Trastuzumab therapy after asymptomatic ejection fraction (EF) decline. We conducted a pooled meta-analysis for the subsequent cardiac events, cardiac mortality, and all-cause mortality. We assessed the quality of included studies using the Newcastle–Ottawa Scale and ROBIN-1 tool. A total of eight cohort studies (six retrospective and two prospective), comprising 222 patients, were found eligible and were included in our analysis. The pooled incidence of cardiac events, cardiac-related mortality, and all-cause mortality was 18% (95% CI 13% to 24%), 5% (95% CI 2% to 10%), and 8% (95% CI 2% to 28%), respectively. The incidence of symptomatic or severe cardiac events was lower in those who received cardioprotective medications concomitant with Trastuzumab. Most patients received either angiotensin-converting-enzyme inhibitors, beta-blockers, or a combination of both. Continuation of planned Trastuzumab therapy with concomitant use of cardioprotective medications can be a safe and effective approach for breast cancer control in patients with asymptomatic EF decline.

**Graphical Abstract:**

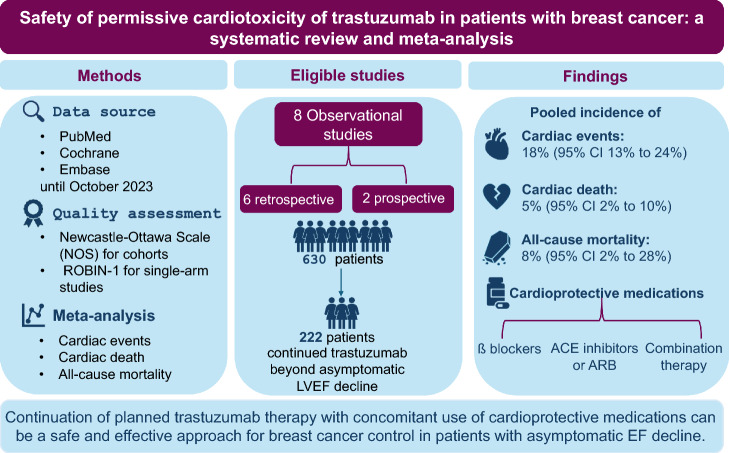

**Supplementary Information:**

The online version contains supplementary material available at 10.1007/s12012-025-10037-z.

## Introduction

There are over two million new breast cancer cases recorded globally [1, 2]. Despite the recent therapeutic advances, breast cancer remains one of the top causes of cancer deaths in women worldwide. Around 15% of women diagnosed with breast cancer have overexpression of the human epidermal growth factor 2 (HER2) [3]. Trastuzumab is a monoclonal antibody targeting HER2 transmembrane receptors and inhibiting the dimerization of HER2. Trastuzumab has revolutionized the treatment landscape for HER2-positive breast cancer, both in the adjuvant and metastatic settings [4–7].

Cardiotoxicity is a well-recognized adverse effect associated with Trastuzumab and the most dose-limiting factor leading to its contraindication in patients with a baseline left ventricular ejection fraction (LVEF) below 50% [8–10]. Permissive cardiotoxicity refers to the intentional acceptance of mild cardiovascular side effects to enable optimal cancer treatment, prioritizing oncologic efficacy while carefully managing cardiac risk [12]. Although this approach has proven feasible**—**for example, in allowing continued Trastuzumab therapy despite minor cardiac effects**—**its long-term outcomes remain uncertain [11, 12]. In addition, patients who have received anthracycline-based chemotherapy, known for its own cardiotoxic effects, are at even higher risk of getting Trastuzumab-induced cardiac dysfunction (TICD) when subsequently treated with Trastuzumab [13]. In patients receiving conventional chemotherapy alongside Trastuzumab, the incidence of symptomatic heart failure ranges from 0.8% to 3.3%, compared to 0.45% in those treated with chemotherapy alone [14, 15]. Asymptomatic reductions in left ventricular ejection fraction (LVEF) have been observed in 2.4% to 7.2% of cases, with some studies reporting incidences as high as 27% [14, 16]. Trastuzumab toxicity commonly manifests within the treatment year [17].

However, most of such cardiac dysfunctions are asymptomatic and reversible after stopping Trastuzumab. This fact, in addition to the desperate need of the life-saving anti-HER2 therapies in many patients with advanced disease, have made many physicians take the risk of giving Trastuzumab-based therapies to either patients with initially low LVEF or for those who suffer asymptomatic decline of LVEF after prior Trastuzumab therapy, an approach named “permissive cardiotoxicity”.

To the best of our knowledge, this is the first comprehensive systematic review of literature that aims to investigate the effect of continuation of Trastuzumab in patients with breast cancer, who developed asymptomatic declines of LVEF.

## Methods

### Search Strategy and Eligibility Criteria

**Fig. 1:**
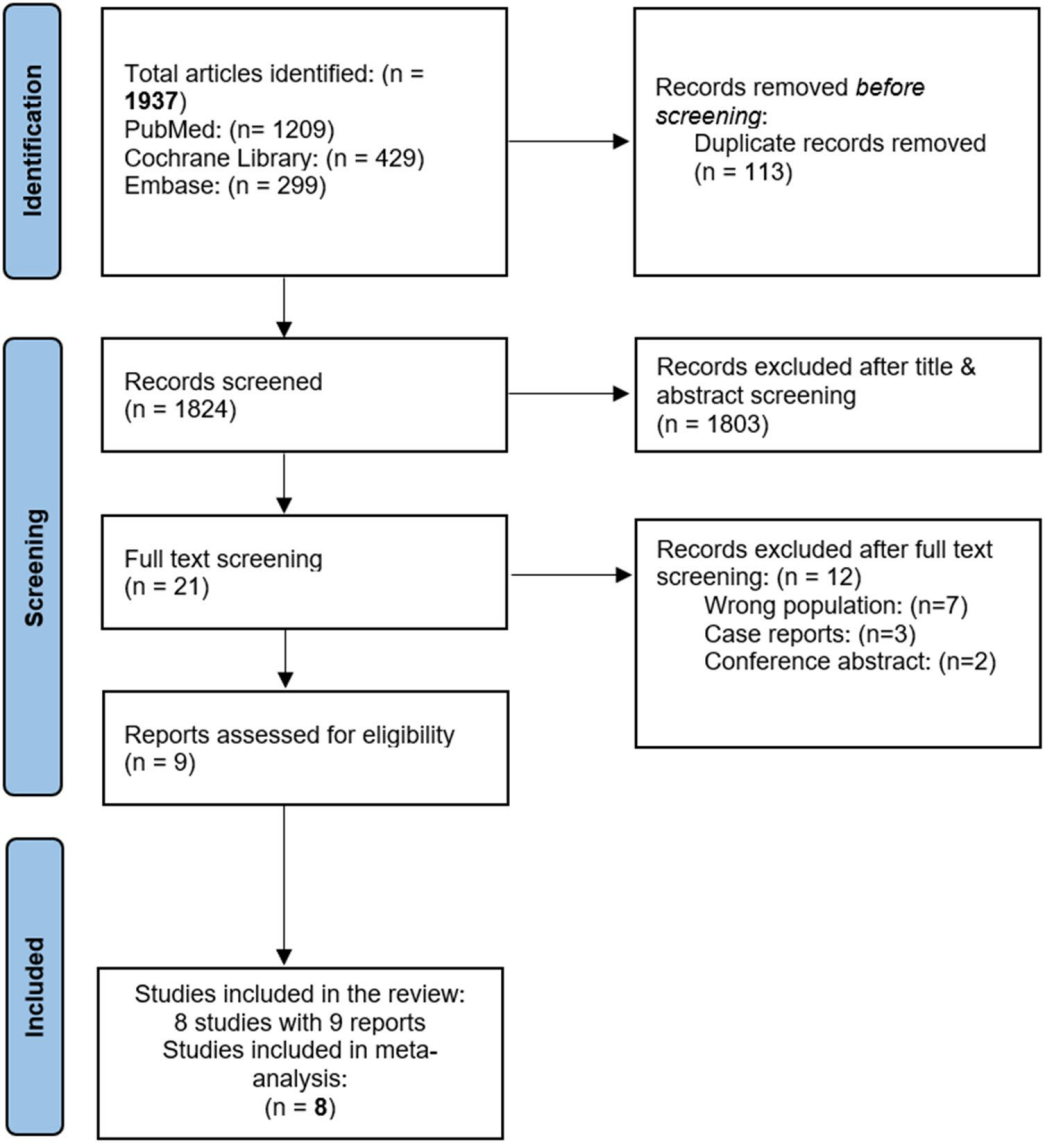
The PRISMA flow diagram of study selection for systematic review on the safety of permissive cardiotoxicity of Trastuzumab

We conducted a systematic search by title and abstract on the following databases: Medline (through PubMed), Cochrane, and Embase until 21 October 2023. We used the MeSH terms to retrieve the synonyms of our search strategy, and the terms were combined using “OR” and “AND” Boolean operators, in accordance with the Cochrane Handbook for Systematic Reviews [18]. The terms were as follows: (Trastuzumab OR Herceptin OR Trazimera OR Trastuzumab-qyyp) AND (Cardi* AND (dysfunction OR toxicit* OR outcome* OR impairment)) AND (breast AND (Cancer OR Tumor OR Neoplasm)) (**Supplementary Table 1)**. Moreover, we reviewed the reference lists of retrieved articles to complement the broad search. We performed this study in adherence to the Preferred Reporting Items for Systematic Reviews and Meta-Analyses (PRISMA) guidelines [19], as shown in Fig. 1**,** and its protocol was registered on PROSPERO (CRD42023489187).

The inclusion criteria for our analysis were as follows:

a. Prospective or retrospective studies conducted on human subjects and published in the English language.

b. The study had to report cardiac outcomes of Trastuzumab-based therapy in patients with HER2-positive breast cancer.

c. The studies must include patients with compromised cardiac function (either at baseline or after prior anti-HER2 therapies.

Case reports and studies with incomplete data were excluded.

### Study Selection and Data Extraction

Two reviewers individually assessed the titles and abstracts of all articles based on predetermined inclusion criteria, and they individually examined the full-text papers for eligibility. Following this, the researchers independently extracted data from the studies using a designated Excel form for data extraction. The final determination of whether to include each paper was reached through consensus among all researchers. Specific data were extracted from the included studies, including study characteristics, cardiac events, cardiac death, left ventricular ejection fraction events, and time to cardiotoxicity. To maintain the integrity of our review and prevent any duplication of data, we conducted a rigorous cross-checking process for the references and data of each study included.

### Critical Appraisal Tool and Risk of Bias Assessment

Two authors independently conducted a quality assessment of the included studies. The authors were blinded to the names and affiliations of the original authors. For observational studies, the assessment utilized the Newcastle–Ottawa Scale (NOS) star scoring system [20]. A score ranging from 7 to 9 stars indicates good quality, 5 to 6 stars is considered moderate quality, and a score of 0 to 4 stars is indicative of poor quality. For single-arm studies, we used the ROBIN-1 tool, which encompasses seven domains. This framework identifies potential sources of bias within a Non-Randomized Study of Interventions (NRSI) [21]. Any disagreements in the quality assessment were resolved by the third independent investigator.

### Statistical Analysis

Statistical analysis was conducted using the R programming language and RStudio software version 4.3.2. Heterogeneity of the included studies was assessed using the Cochrane Q test, and heterogeneity was presented as I^2^. Pooled prevalence of cardiac events, cardiac deaths, and all-cause mortality was assessed using the pooled proportion calculation. The fixed effects model was used for cardiac events and cardiac deaths, while the random effect model was used for all-cause mortality because of significant heterogeneity with p value < 0.05. In addition, we performed a sensitivity analysis using the leave-one-out model when we detected significant heterogeneity among the pooled studies. Given that our meta-analysis included only eight studies, the statistical power of Eggers’ or Peters’ test may not be high enough to detect real asymmetry because it is generally recommended to perform a test when our meta-analysis includes at least 10 studies [22, 23]. Therefore, we relied on visual inspection of the funnel plots to assess the risk for publication bias.

## Results

### Summary of the Included Studies

Our database search identified a total of 1937 studies. Among which, 1916 articles were excluded, and 21 full-text articles were evaluated for eligibility. Following the full-text screening, nine articles with eight studies were found eligible and were included in our analysis (Fig. 1). Six were retrospective, and two were prospective studies with a total of 630 patients, among whom 222 continued Trastuzumab beyond asymptomatic LVEF decline. The median age of included patients ranged from 52.6 to 57.4 years, and the median follow-up ranged from 2.5 to 58.5 months. Trastuzumab was the used anti-HER2 agent in all the studies except one that allowed Trastuzumab, dual HER2 blockade, or T-DM1. Patients were included with low baseline LVEF in 3 studies, after decline of LVEF on prior anti-HER2 therapy in 4 studies, or with both indications allowed in one study. The baseline LVEF values across included studies indicate a range of cardiac functions among breast cancer patients receiving Trastuzumab therapy. Barron et al. 2019 found a mean baseline LVEF of 59.3%, and Hussain et al. 2019 reported a mean of 59%. Bonardeaux et al. 2023 observed a higher baseline LVEF of 61.6%. In contrast, Nowsheen et al. 2018 reported a mean LVEF of 45.4% in patients with low LVEF, suggesting more compromised cardiac function. Leong et al. 2019 reported a mean baseline LVEF of 49%, while Lynce et al. 2019 documented an even lower mean of 45%. Khoury et al. 2021 reported a mean baseline LVEF of 44.9%. Additionally, Bouwer et al. 2022 observed a mean LVEF of 46%, and Zhou et al. 2023 reported a baseline LVEF of 48%. The baseline characteristics of the patients are summarized in Table 1.

**Table 1:** Summary of the baseline characteristics of the included studies

Study ID	Study design	Number of patients	Age Mean ± SD	Age Mean ± SD	Indication for inclusion	Mean LVEF	Cardioprotective drugs prescribed	Mean LVEF at follow-up	Cancer stage
Nowsheen et al. 2018 [24]	Retrospective	Total (n = 428) Normal LVEF (n = 408) Low LVEF (n = 20)	NA	NA	Baseline LVEF < 53%	Normal LVEF (63.4) Low LVEF (45.4)	NA	NA	Most patients presented with node-positive disease, only a minority had metastasis
Barron et al. 2019 [25]	Retrospective	18	57.4 ± 10.5	57.4 ± 10.5	LVEF decline on Trastuzumab	59.3 ± 4.84	BB (n = 13) ACE-I (n = 10) ARB (n = 2)	55.1 ± 3.24	Most patients had early-stage disease; two patients had metastasis
Hussain et al. 2019 [26]	Retrospective	23	53.7 ± 9.9	53.7 ± 9.9	LVEF decline on Trastuzumab	59 ± 6.32	BB (n = 5) ACE/ARB (n = 3)	43 ± 6.32	Early-stage group (n = 14) Metastatic group (n = 9)
Leong et al. 2019 [27]	Prospective	20	59 ± 11	59 ± 11	LVEF decline on Trastuzumab or baseline LVEF 40–54%	49 ± 2	ACE-I or ARB or BB (n = 15) both (n = 25)	51 ± 2	All patients had early-stage disease; no patients had metastasis
Lynce et al. 2019 [28]; Khoury et al. 2021 [29]	Prospective Phase I	30 (23 evaluable)	53.6 ± 12.5	53.6 ± 12.5	Baseline LVEF 40–49%	44.8 ± 2.6	BB (n = 27) ACE-I (n = 21)	45.7 ± 6.3	Early-stage group (n = 18) Metastatic group (n = 13)
Bouwer et al. 2022 [30]	Retrospective	37	52.6 ± 13.8	52.6 ± 13.8	Baseline LVEF 40–49%	46 ± 3.08	BB (n = 6) ACE (n = 7) both (n = 8)	42 ± 6.17	All patients had metastasis
Bonardeaux et al. 2023 [31]	Retrospective	23	56 ± 10	56 ± 10	LVEF decline on Trastuzumab	61.6 ± 4.1	BB (n = 14) ACE (n = 11) ARB (n = 6)	57.2 ± 6.5	Early-stage group (n = 17) Metastatic group (n = 6)
Zhou et al. 2023 [11]	Retrospective	51	56 ± 11	56 ± 11	LVEF decline on Trastuzumab	48 ± 4	ACE-I or ARB (n = 49) BB (n = 43) Both (n = 43)	57.5 ± 4	Seven patients had metastasis

*LVEF*, Left ventricular ejection fraction, *SD* Standard deviation, *HER-2* Human epidermal growth factor receptor 2, *BB* Beta-blockers, *ACE-I* Angiotensin converting enzyme inhibitors, *ARB* Angiotensin receptor blockers, *NA* Not applicable

### Quality Assessment

**Fig. 2:**
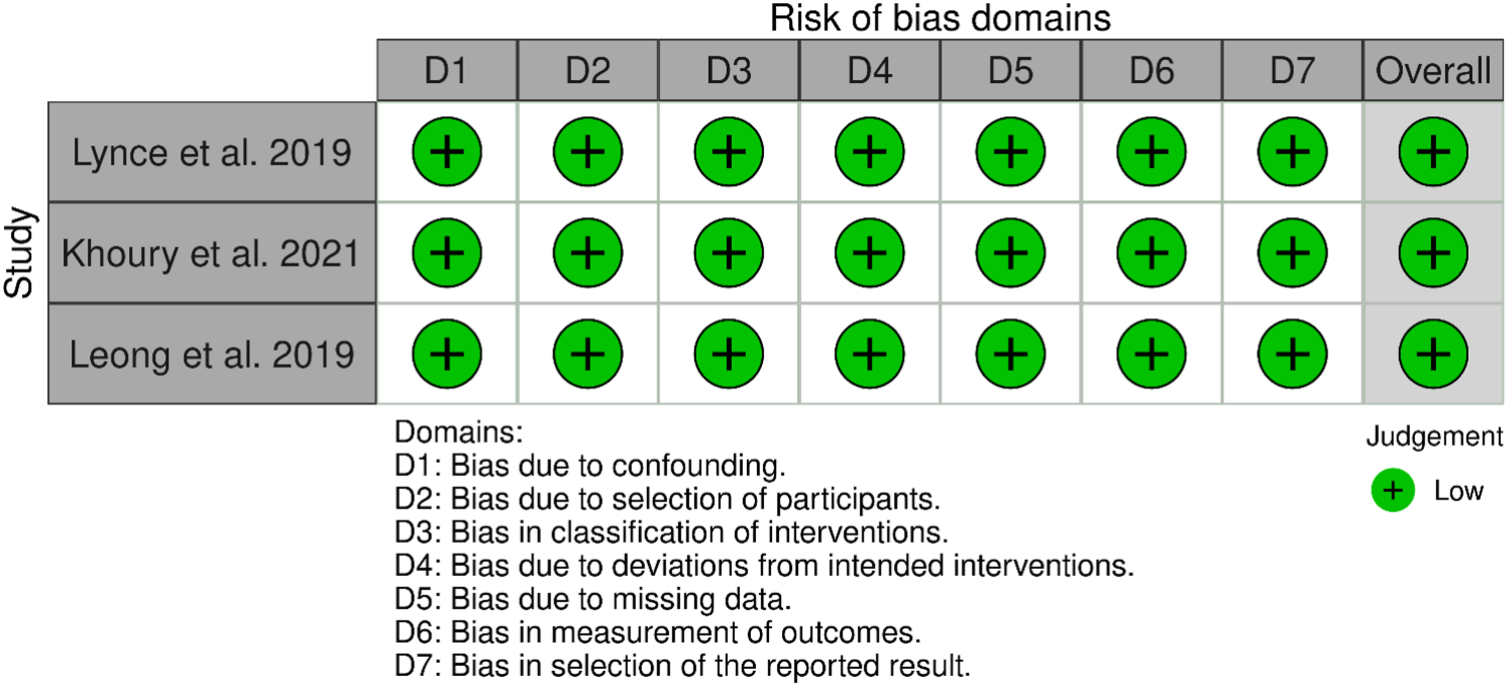
Quality assessment of the included single-arm studies using ROBIN Quality Assessment tool

We assessed the quality of the included cohort and prospective studies using the new castle Ottawa scale (NOS) and ROBIN Quality Assessment tools. Our included cohort and prospective studies were rated as good or fair quality (Table 2**, **Fig. 2).

**Table 2:** Quality assessment of the included cohort studies using the new castle Ottawa scale

Study ID	Newcastle Ottawa scale assessment domains for cohort studies								
	Selection				Comparability	Outcome			
	Representativeness of the exposed cohort	Selection of the non-exposed cohort	Ascertainment of exposure	Demonstration of the outcome of interest was not present at start of study	Comparability of cohorts based on the design or analysis	Assessment of outcome	Was follow-up long enough for outcomes to occur	Adequacy of follow-up of cohorts	Quality Score
Barron et al 2019 [25]	NA	NA	*	*	**	*	*	**	Fair
Bouwer et al 2022 [30]	NA	NA	**	*	**	*	*	**	Good
Bonardeaux et al 2023 [31]	NA	*	*	*	**	*	*	**	Good
Hussain et al 2019 [26]	NA	NA	*	*	**	*	*	**	Fair
Nowsheen et al 2018 [24]	*	*	*	*	**	*	*	**	Good
Zhou et al 2023 [11]	NA	NA	*	*	**	*	*	**	Fair

Abbreviations: *NA* Not applicable for the study design or methodology

### Cardiotoxicity During Cancer Treatment

**Fig. 3:**
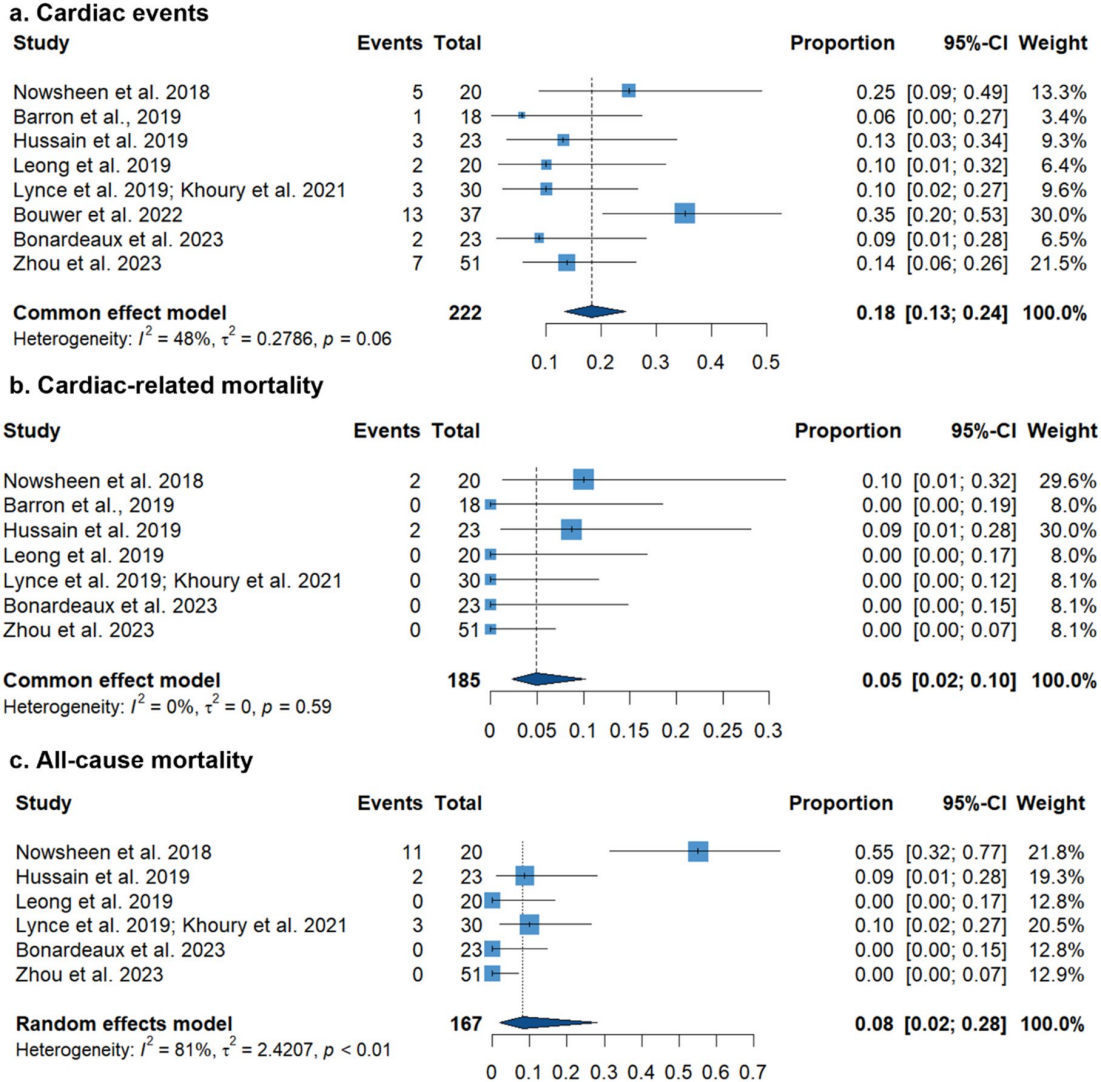
Forest plot showing pooled incidence of (**a**) Cardiac events, (**b**) Cardiac-related mortality, and (**c**) All-cause mortality

The pooled prevalence of cardiac events among the included studies was 18% (95% CI 13% to 24%), (low heterogeneity; I^2^ = 48%) (Fig. 3A). In the six studies that reported time to events, the mean time to subsequent cardiac events ranged from 10.5 to 60.8 weeks. In the three studies that reported cardiac outcomes that received ACEIs, ARBs, and/or BBs, Khoury et al. (2021) and Hussain et al. (2019) both reported a lower cardiac event rate of 8.7–13%. Out of the reported cardioprotective medications prescribed, 108 patients received beta blockers, 109 received ACE inhibitors or ARB, and 76 received combination therapy. Of note, the reporting of the use of cardio-protective agents was neither consistent nor homogenous across the included studies (Table 3).

**Table 3:** Summary of cardiac outcomes from the included studies

Study ID	Cardiac events	Mean Time to cardiotoxicity (weeks)	LVEF before resuming TZT	Mean LVEF at follow-up	Cardiac death	all-cause death
Nowsheen et al. 2018 [24]	5/20 (25%)	60.8	Normal LVEF (53) Low LVEF (25)	NA	2	11
Barron et al. 2019 [25]	1/18 (5.56%)	NA	59.3 ± 4.84	55.1 ± 3.24	0	NA
Hussain et al. 2019 [26]	3/23 (13.04%)	16	55.5	43 ± 6.32	2	2
Leong et al. 2019 [27]	2/20 (10%)	10.5	40	51 ± 2	0	0
Lynce et al. 2019 [28]; Khoury et al. 2021 [29]	3/30 (10%)	32.71	40	45.7 ± 6.3	0	3
Bouwer et al. 2022 [30]	13/37 (35.14%)	28	44	42 ± 6.17	NA	NA
Bonardeaux et al. 2023 [31]	2/23 (8.7%)	NA	61.6 ± 4.1	57.2 ± 6.5	0	NA
Zhou et al. 2023 [11]	7/51 (13.73)	22.14	48 ± 4	57.5 ± 4	0	0

*LVEF* Left ventricular ejection fraction, *TZT* Trastuzumab, *NA* Not applicable

**Fig. 4:**
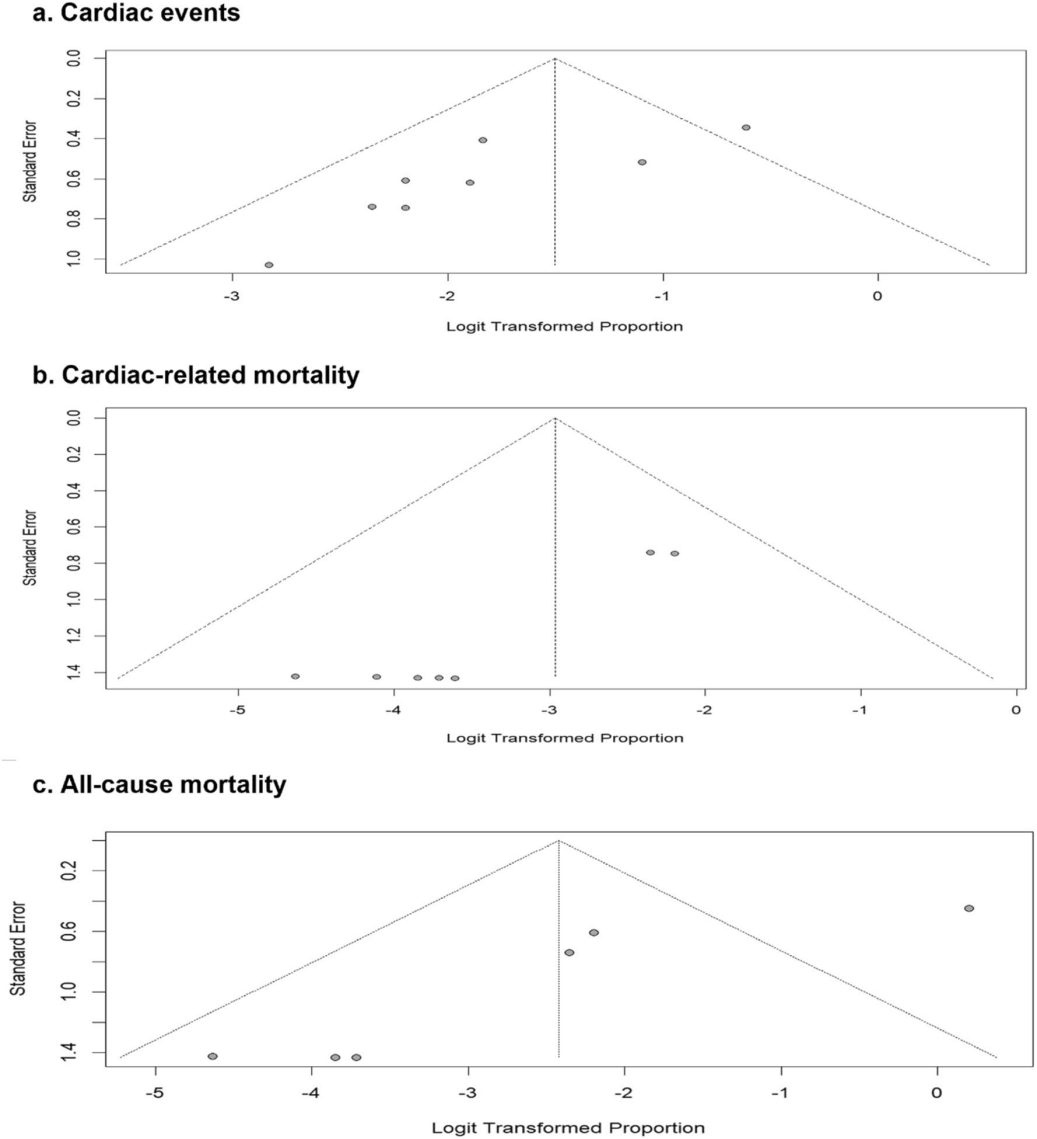
Funnel plot for detection of publication bias in (**A**) Cardiac events, (**B**) Cardiac-related mortality, and (**C**) All-cause mortality

The incidence of cardiac mortality was reported in eight studies, with a pooled incidence of only 5% (95% CI 2% to 10%), (low heterogeneity; I^2^ = 0%). The pooled incidence of all-cause mortality was 8% (95% CI 2% to 28%), (significant heterogeneity; I^2^ = 81%) (Fig. 3B and 3 C). This significant heterogeneity was resolved after performing the leave-one-out sensitivity analysis omitting Nowsheen et al. 2018 (I^2^ = 0%), with a pooled all-cause mortality of 6% (95% CI 3% to 13%). Finally, visual inspection of the funnel plots for all outcomes showed asymmetry, which suggests the presence of publication bias (Fig. 4).

## Discussion

In our meta-analysis and systematic review, we offer crucial insights into the outcomes of patients with HER2-positive breast cancer who continue anti-HER2 therapy after experiencing asymptomatic left ventricular function decline. Our analysis shows a low cardiac mortality rate and a relatively low subsequent significant cardiac event rate, which should guide the clinical decisions in patients when continuing the life-saving anti-HER2 therapy.

Anticancer treatments are associated with two types of myocardial dysfunction: type I, linked to anthracyclines, which leads to irreversible myocyte destruction, and type II, associated with Trastuzumab and other treatments, characterized by myocyte dysfunction without cell death [17]. Type II dysfunction is not dose-dependent, lacks ultrastructural changes allowing potential recovery after a few months, and often permits treatment reintroduction after recovery. Both types of cardiotoxicities can coexist in patients undergoing concomitant or sequential chemotherapy with anti-HER2 [17].

A review of three adjuvant trials of Trastuzumab that included a total of 7445 patients showed that despite the beneficial effects of Trastuzumab, there is evidence of an increased risk of developing cardiac events in 8.7% patients and severe congestive heart failure (CHF) in 2.3% of cases that led to treatment termination in 10% of cases, even if 76.2% of patients could be treated to completion and traditional prognostic determinants included LVEF < 60%, hypertension, BMI > 25, age >  = 60 and ethnic minorities [7]. Trastuzumab cardiotoxicity is usually characterized by a decrease in LVEF and results in the development of heart failure (HF) in about 0–4.1% of treated patients [32–34]. However, no cardiac deaths are usually observed, and a significant proportion of patients do not develop severe cardiac events or cardiotoxicity [28–30]. Such a usual mild course of cardiac events, together with the major impact of anti-HER2 therapies on the patients’ survival, introduces the concept of permissive cardiotoxicity. In addition to Trastuzumab, several factors can contribute to EF decline in cancer patients, such as pre-existing cardiovascular conditions, prior or concurrent use of anthracyclines, radiation therapy, age, comorbidities, and lifestyle factors like smoking or inactivity [35–38].

The use of cardiac medications, such as beta-blockers, ACE inhibitors, and angiotensin II receptor blockers (ARBs), has also been shown to improve left ventricular ejection fraction (LVEF) [27, 39, 40]. In the study by Barron et al., 18 patients experienced a reduction in LVEF yet opted to continue Trastuzumab therapy; the use of angiotensin-converting enzyme inhibitors and beta-blockers resulted in an elevation in LVEF by an average of 4.6% points over time (95% CI 1.9–7.4) and approached initial baseline values. Consequently, 94% of the patients remained asymptomatic in subsequent follow-ups, and no deaths occurred, suggesting a potential protective or restorative effect of these drugs in the context of Trastuzumab-induced cardiotoxicity [25]. Indeed, in the 2022 ESC guidelines, an algorithm for managing Trastuzumab-induced cardiotoxicity is offered, stating the need for regular cardiac evaluation, as well as the initiation of beta-blocker and ACE-inhibitor therapy in patients with an asymptomatic decline in LVEF. These guidelines are consistent with the observation in our study that patients who continued to receive Trastuzumab treatment along with these cardio-protective agents had significantly lower rates of severe cardiac adverse events without any increase in cardiac deaths, thus justifying the concept of permissive cardiotoxicity in HER2-positive breast cancer management [41, 42].

In our analysis, the pooled incidence of subsequent cardiac events was 18%, and the cardiac mortality rate was 5%. In addition, and despite including metastatic patients in some of the studies, the all-cause mortality rate was 8% in patients who adopted the permissive cardiotoxicity approach; however, the high heterogeneity of the included studies (I^2^ 81%) may require caution while interpreting these findings. These data should be incorporated when weighing the benefit of continuing anti-HER2 therapies versus the risk of further cardiotoxicity. In the studies included in our systematic review, most of the patients could complete the planned course of Trastuzumab.

It is also important to identify patients with a higher risk of developing severe cardiotoxicity, in which various echocardiographic parameters, such as LVEF before initial chemotherapy, baseline LVEF before treatment with Trastuzumab, and initial global longitudinal strain, could be reliable predictors [31]. In addition, other conventional cardiac risk factors have been proven to increase the risk of Trastuzumab-induced cardiotoxicity in a systematic review and meta-analysis by Koulaouzidis et al. In this analysis, age, smoking, hypertension, diabetes, and personal or family history of ischemic heart disease were associated with increased risk of TIC [43]. Also, of considerable value is the identification of patients who are likely to face an increased risk of cardiotoxicity. The HFA-ICOS baseline cardiotoxicity risk score includes parameters such as age, hypertension, diabetes, pre-existing cardiovascular disease, previous anthracycline exposure, and baseline LVEF, all of which have been established to confer relative cardiotoxicity at different levels for patients being treated with Trastuzumab [17]. Such factors must be critically evaluated regarding the continuation of Trastuzumab in very high-risk patients. Indeed, such parameters should be taken into consideration when deciding to allow permissive cardiotoxicity.

Management of patients with Trastuzumab-induced asymptomatic LVEF decline remains controversial. Several patients with LVEF below 50% could tolerate additional Trastuzumab doses with concurrent cardiac medication, which suggests a potential strategy for continuing effective cancer therapy while managing cardiac risk. However, this approach requires careful clinical judgment and close monitoring. In addition, the long-term cardiac outcomes in patients treated with Trastuzumab are critical [44]. Most patients showed recovery in left ventricular LV function post-treatment, but a small percentage experienced persistent cardiotoxicity over a three-year period [45]. This finding indicated the need for extended cardiac follow-up in some patients, even after the completion of cancer therapy.

While our analysis provides a detailed examination of permissive cardiotoxicity in HER2-positive breast cancer patients treated with Trastuzumab, it is limited by the heterogeneity of the included studies and its primary focus on short-term outcomes, thereby restricting the generalizability of findings to long-term cardiotoxic effects. Also, it is limited by the small number of studies included, indicating a need for additional trials to validate these findings. Despite the asymmetry of funnel plots for all outcomes, we couldn’t assess the risk for publication bias using Egger’s or Peters’ tests due to the small number of included studies. Finally, the concomitant cardioprotective therapies used were heterogeneous and not adequately reported in many studies.

## Conclusion

In conclusion, continuation of planned Trastuzumab therapy among patients with low risk for development of severe cardiac events may be a safe and effective approach in patients with high-risk breast cancer where the continuation of anti-HER2 therapy carries substantial improvement in survival. However, further prospective studies are required to accurately assess the rate of severe cardiotoxicity and cardiac-related deaths among patients adopting this approach.

## Supplementary Information

Below is the link to the electronic supplementary material.

Supplementary file1 (DOCX 2947 KB)

## Data Availability

The datasets used and/or analyzed during the current study are available from the corresponding author upon reasonable request.
