# Supplementary material for: Safety of Permissive Cardiotoxicity of Trastuzumab in Patients with Breast Cancer: A Systematic Review and Meta-Analysis
Source: Cardiovasc Toxicol. 2025 Jul 8;25(9):1369–80. doi: 10.1007/s12012-025-10037-z (PMC12310907; doi:10.1007/s12012-025-10037-z)
Supplement: Supplementary file 1 — Supplementary file1 (DOCX 2947 KB) [file 12012_2025_10037_MOESM1_ESM.docx]

**Supplementary materials**

**Supplementary Table 1** Detailed search strategy for each database

| **Database** | **Search Terms** | **Search Field** | **Search Results** |
| --- | --- | --- | --- |
| PubMed | (Trastuzumab OR Herceptin OR Trazimera OR Trastuzumab-qyyp) AND (Cardi* AND (dysfunction OR toxicit* OR outcome* OR impairment)) AND (breast AND (Cancer OR Tumor OR Neoplasm)) | All Field | 1209 |
| Cochrane Library | (Trastuzumab OR Herceptin OR Trazimera OR Trastuzumab-qyyp) AND (Cardi* AND (dysfunction OR toxicit* OR outcome* OR impairment)) AND (breast AND (Cancer OR Tumor OR Neoplasm)) | All Field | 429 |
| Embase | (Trastuzumab OR Herceptin OR Trazimera OR Trastuzumab-qyyp) AND (Cardi* AND (dysfunction OR toxicit* OR outcome* OR impairment)) AND (breast AND (Cancer OR Tumor OR Neoplasm)) | Title and abstract | 299 |
